# Low‐grade gangliogliomas in adults: A population‐based study

**DOI:** 10.1002/cam4.3577

**Published:** 2020-10-27

**Authors:** Xiaoning Lin, Rong Huang, Pengfei Zhang, Jin Sun, Guijiang Dong, Yanlin Huang, Xinhua Tian

**Affiliations:** ^1^ Department of Neurosurgery Zhongshan Hospital Xiamen University Xiamen China; ^2^ Department of Child Health, Women and Children's Hospital Xiamen University Xiamen China; ^3^ Institute of Molecular Immunology School of Laboratory Medicine and Biotechnology Southern Medical University Guangzhou China

**Keywords:** adults, low‐grade ganglioglioma, prognostic factor, SEER, treatment

## Abstract

**Background:**

Low‐grade gangliogliomas (GGs) are rare tumors of the central nervous system in adults. This study aims to define their characteristics, prognostic factors, and the impact of different treatment patterns on survival.

**Methods:**

The Surveillance, Epidemiology, and End Results (SEER) database was used to investigate the potential clinicopathological factors of low‐grade GGs in adult patients (age ≥18 years). Kaplan–Meier method and Cox regression model were utilized to evaluate the associations between variables and overall survival (OS).

**Results:**

A total of 703 adult patients diagnosed with low‐grade GGs were identified between 2004 and 2016, with a median follow‐up period of 60.0 months. The median age at diagnosis was 32.0 years, with 50.1% of patients being male, 84.2% white people, and 40.2% of married status. The predominant tumor site was located in temporal lobe (38.8%). The median OS time for the whole cohort was not reached. The 5‐ and 10‐year OS rates for patients underwent gross total resection (GTR) were 92.5% and 87.2%, respectively. Univariate and multivariate analysis showed age, gender, tumor site, and treatment pattern were significant factors for OS. The employment of adjuvant radiotherapy (RT) and/or chemotherapy would significantly shorten OS time.

**Conclusions:**

This is the largest retrospective study of adult low‐grade GGs up to date. Younger age, female gender, temporal lobe location, and GTR indicated better survival. Adjuvant RT and/or chemotherapy should not be considered after whatever surgery in adult patients with low‐grade GGs, unless the malignant transformation has been confirmed.

## Graphical abstract

1. Age, gender, tumor site, and treatment pattern are important factors for overall survival, and the best treatment is gross total resection whenever safely possible.
2. Adjuvant radiotherapy and/or chemotherapy may have a negative impact on patients' overall survival.

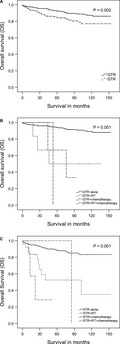

## 1 INTRODUCTION

Gangliogliomas (GGs) are relatively rare, low grade, and slowly growing neuroepithelial neoplasms, accounting for only 0.4% of all central nervous system (CNS) tumors and 1%–7.6% of primary brain tumors.^1^, ^2^, ^3^, ^4^ Histopathological examination exhibits ganglionic and glial cells components, both of which are thought to originate from glioneuronal precursor cells.^4^ According to the World Health Organization (WHO) classification of brain tumors, GGs are classified as grade I, also referred to as low‐grade tumors.^5^ The highest incidence rates are found in children and young adults, with a slight male predominance.^6^, ^7^, ^8^ These tumors can be located anywhere within the CNS, however, predominantly in the temporal and frontal lobes, and therefore, usually associated with chronic epilepsy.^9^, ^10^

Tumor resection is the standard treatment of choice for GGs, and a gross total resection (GTR) is the prime objective for both low‐grade and high‐grade GGs, because it has been demonstrated to significantly control seizures and prolong survival.^11^, ^12^, ^13^ For adults with subtotally resected tumors (STR), adjuvant radiation therapy (RT) and chemotherapy will be considered, however, the definitive efficacy of which still remains controversial, especially in low‐grade GGs.^3^, ^11^, ^14^, ^15^, ^16^ Some studies show that RT can only improve the local control but not the overall survival (OS) of tumors with any grade.^11^ Similarly, the impact of chemotherapy is also inconclusive due to the paucity of research data.^3^, ^14^ In addition, because of the rarity of GGs, it is still difficult to perform prospective studies now or the near future.

In the present study, we aim to report the largest series of adult patients with low‐grade GGs based on the Surveillance, Epidemiology, and End Results (SEER) database. To better understand these rare tumors, our study investigated the epidemiology, prognostic factors, and in particular, the impact of different treatment patterns on survival, to further define the role of adjuvant therapies in low‐grade GGs.

## 2 MATERIALS AND METHODS

### 2.1 Study population

The data information for this study was extracted from the recent SEER program (www.seer.cancer.gov) (1975–2016), which is maintained by the National Cancer Institute, Division of Cancer Control and Population Sciences, Surveillance Research Program, Surveillance Systems Branch (released April 2019, based on the November 2018 submission). We included data from the incidence SEER 18 registries custom data (with additional treatment fields).

### 2.2 Inclusion criteria, exclusion criteria, and data collection

Only adult patients (age ≥18 years) with a pathologically confirmed ganglioglioma and gangliocytoma (International Classification of Diseases for Oncology, 3rd Edition histology codes 9505/0, 9505/1, and 9492/0) between 1 January 2004 and 31 December 2016 were included in the current study. Patients with anaplastic ganglioglioma (9505/3), unknown survival time, unknown surgery (code 90 and 99), and unknown radiation record were excluded from the present study. Data collected for analysis included age at diagnosis, gender, race, marital status, tumor site, extent of surgical resection, adjuvant treatment (RT and chemotherapy), and OS.

### 2.3 Data analysis and statistical methods

We categorized age as 18–29, 30–39, 40–49, 50–59, 60–69, and >69 years. Race was categorized into white, others (including black and American Indian/Alaska Native or Asian/Pacific Islander), and unknown. Tumor site was recorded in 15 categories, including the various brain lobes, as well as cerebellum, cerebrum, ventricle, brain stem, spinal cord, pituitary gland, pineal gland, cerebral meninges, optic nerve, brain‐not otherwise specified (NOS), and overlapping lesion of brain. The extent of surgical resection was divided into three groups, including GTR, STR, and biopsy on the basis of SEER surgery codes guidelines and other previous study.^15^ For further survival analysis, we categorized age as “<40 years” and “≥40 years,” tumor site as “temporal lobe,” “frontal lobe,” and “other sites,” according to the previous literatures.^3^, ^11^

Baseline patient characteristics were summarized by standard descriptive statistics and frequency tabulation. Overall survival analysis was estimated by using the Kaplan–Meier method and compared with the log‐rank test. Univariate and multivariate Cox regression models were applied to evaluate the effect of variables of interest on OS. All statistical analyses were performed in SPSS software version 25.0 (IBM Corp., Armonk, NY, USA), and *p* < .05 was considered statistically significant. Ethical approval or informed consent was waivered for this study because of the fully anonymized information of the patients included in the SEER.

## 3 RESULTS

### 3.1 Clinical characteristics

A total of 703 adult patients diagnosed with low‐grade GGs were identified between 2004 and 2016. Of the whole population, the mean and median ages at diagnosis were 36.0 and 32.0 years, respectively, with 50.1% of patients being male, 84.2% white people, and 40.2% of married status (Table 1). Regarding tumor site, 273 patients (38.8%) had temporal lobe tumors, followed by frontal (14.7%), and parietal lobe (9.5%) (Table 2).

**TABLE 1:** Clinical characteristics of adult patients with low‐grade GGs.

Characteristic	n (%)
Age at diagnosis (y)	
18–29	298 (42.4)
30–39	152 (21.6)
40–49	116 (16.5)
50–59	77 (10.9)
60–69	42 (6.0)
>69	18 (2.6)
Gender	
Female	351 (49.9)
Male	352 (50.1)
Marital status	
Unmarried	371 (52.8)
Married	283 (40.2)
Unknown	49 (7.0)
Race	
White	592 (84.2)
Others	99 (14.1)
Unknown	12 (1.7)
Tumor site	
Temporal lobe	273 (38.8)
Frontal lobe	103 (14.7)
Other sites	327 (46.5)
Extent of surgical resection	
GTR	385 (54.7)
STR	236 (33.6)
Biopsy	82 (11.7)
Radiotherapy	
No	661 (94.0)
Yes	42 (6.0)
Chemotherapy	
No	688 (97.9)
Yes	15 (2.1)
Vital status	
Alive	624 (88.8)
Dead	79 (11.2)

**TABLE 2:** Tumor sites of adult low‐grade GGs.

Tumor site	n (%)
Temporal lobe	273 (38.8)
Frontal lobe	103 (14.7)
Parietal lobe	67 (9.5)
Cerebellum, NOS	54 (7.7)
Occipital lobe	32 (4.6)
Brain, NOS	32 (4.6)
Ventricle, NOS	30 (4.3)
Cerebrum	29 (4.1)
Overlapping lesion of brain	25 (3.6)
Spinal cord	22 (3.1)
Brain stem	21 (3.0)
Pituitary gland	10 (1.4)
Pineal gland	3 (0.4)
Cerebral meninges	1 (0.1)
Optic nerve	1 (0.1)
Total	703 (100.0)

### 3.2 Treatment strategy

In current study, 54.7% of the patients underwent GTR, 33.6% of the patients underwent STR, and 11.7% had a biopsy only. Due to the small number of patients in the group of biopsy, these patients were included in the cohort undergoing STR for subsequent survival analysis. For adjuvant therapies, RT was used in 6.0% of patients, and 15 patients (2.1%) had chemotherapy (Table 1). In addition, according to the different extent of surgical resection, the group of GTR was subdivided into four groups, including GTR alone (97.4%), GTR + RT (1.5%), GTR + chemotherapy (0.3%), and GTR + RT + chemotherapy (0.8%). Similarly, the group of STR was also subdivided into four groups, including STR alone (88.7%), STR + RT (7.9%), STR + chemotherapy (0.9%), and STR + RT + chemotherapy (2.5%) (Table 3).

**TABLE 3:** Treatment patterns of adult patients with low‐grade GGs.

Treatment	n (%)
GTR	385
GTR alone	375 (97.4)
GTR + RT	6 (1.5)
GTR + chemotherapy	1 (0.3)
GTR + RT + chemotherapy	3 (0.8)
STR	318
STR alone	282 (88.7)
STR + RT	25 (7.9)
STR + chemotherapy	3 (0.9)
STR + RT + chemotherapy	8 (2.5)

### 3.3 Overall survival analysis

The median OS time for the whole cohort was not reached with a median follow‐up time of 60.0 months, and 79 patients (11.2%) died during follow‐up. The 5‐ and 10‐year OS rates for adult patients with low‐grade GGs were 89.2% and 83.1%, respectively. Prognostic factors identified on univariate analysis were age at diagnosis, gender, tumor site, and treatment pattern (Table 4). On further multivariate Cox regression, younger age, female, temporal lobe tumors, GTR, and no adjuvant therapies (RT and/or chemotherapy) were significant factors for longer OS (Table 4).

**TABLE 4:** Univariate and multivariate analysis of OS.

Variable	Univariate analysis		Multivariate analysis	
	HR (95% CI)	Overall *p*‐value	HR (95% CI)	Overall *p*‐value
Age at diagnosis (y)		<0.001		<0.001
<40	Reference		Reference	
≥40	4.990 (3.068–8.116)		4.225 (2.540–7.026)	
Gender		0.023		0.007
Female	Reference		Reference	
Male	1.694 (1.076–2.669)		1.946 (1.204–3.146)	
Marital status		0.529		
Unmarried	Reference			
Married	1.191 (0.750–1.890)			
Unknown	1.526 (0.680–3.424)			
Race		0.976		
White	Reference			
Others	0.948 (0.501–1.795)			
Unknown	0.856 (0.119–6.169)			
Tumor site		<0.001		0.016
Temporal lobe	Reference		Reference	
Frontal lobe	2.657 (1.267–5.575)		2.174 (1.004–4.705)	
Other sites	3.282 (1.817–5.929)		2.410 (1.317–4.411)	
Treatment		<0.001		<0.001
GTR alone	Reference		Reference	
GTR + RT	6.544 (1.988–21.534)		5.074 (1.470–17.523)	
GTR + chemotherapy	16.138 (2.180–119.480)		19.100 (2.333–156.339)	
GTR + RT + chemotherapy	8.680 (2.060–36.569)		4.812 (1.119–20.698)	
STR alone	1.736 (1.035–2.913)		1.440 (0.854–2.427)	
STR + RT	8.225 (3.974–17.023)		4.062 (1.932–8.540)	
STR + chemotherapy	6.034 (0.818–44.494)		8.514 (1.117–64.912)	
STR + RT + chemotherapy	18.189 (6.196–53.393)		8.604 (2.844–26.029)	

**FIGURE 1:**
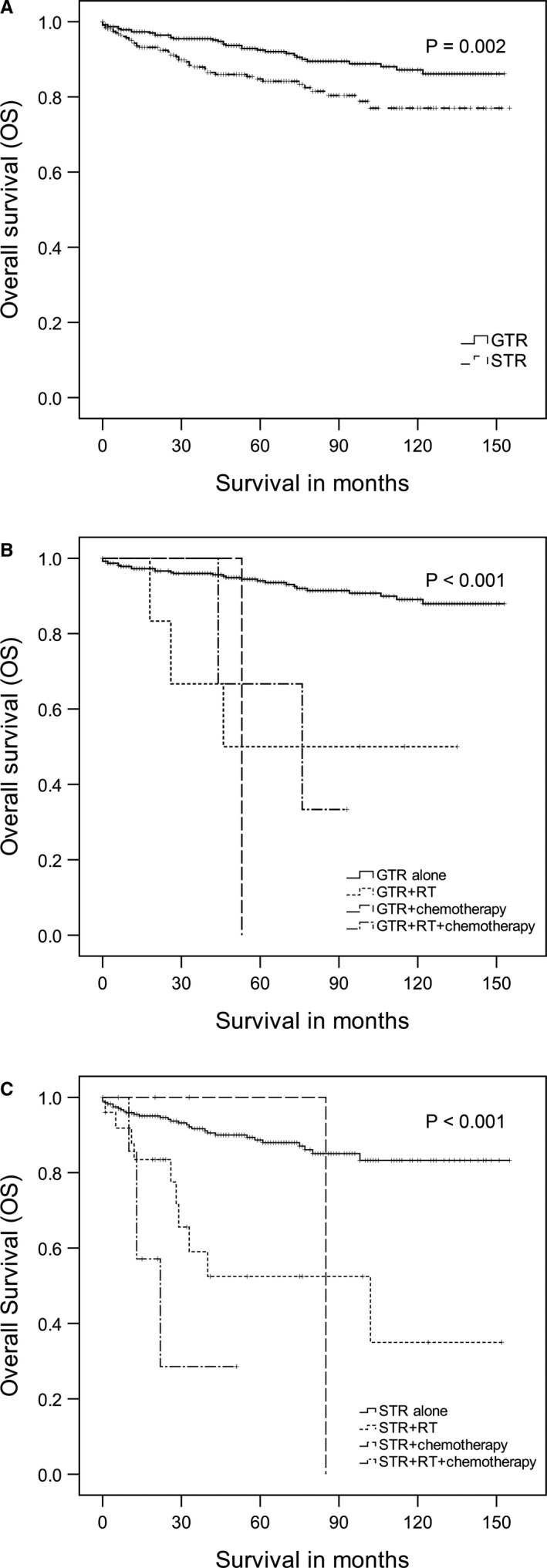
Overall survival of adult low‐grade gangliogliomas. A. The impact of gross total resection (GTR) and subtotal resection (STR) on overall survival (OS). B. The impact of adjuvant radiotherapy (RT) and/or chemotherapy on OS from a subgroup analysis of patients who underwent GTR. C. The impact of adjuvant RT and/or chemotherapy on OS from a subgroup analysis of patients who underwent STR

Because of the possible negative impact of the adjuvant therapies on OS, to better account for the prognosis of these patients who received adjuvant therapies, further analyses were performed in the subgroups of GTR and STR. For the GTR group, the 5‐ and 10‐year OS rates were 92.5% and 87.2%, respectively, however, the 5‐ and 10‐year OS rates for patients in the group of STR were 84.8% and 77.0%, respectively (*p* = 0.002, log‐rank test; Figure 1A). According to the GTR subgroup analysis, compared with other three treatment patterns, GTR alone had the best OS time and the difference was significant (*p* < 0.001, log‐rank test; Figure 1B). The similar results were also found even in the other subgroup of STR, which suggested that the adjuvant therapies, especially RT received, resulted in significantly worse OS than STR alone (*p* < 0.001, log‐rank test; Figure 1C). In addition, we also compared the group receiving adjuvant treatment with the group that was only operated, and the results are showed in Table S1.

## 4 DISCUSSION

Up to date, most studies of low‐grade GGs are individual case reports or include a limited number of patients less than 350.^2^, ^20^ The largest report, which included 402 patients, not only mixed pediatric and adult cases, but also focused on both low‐grade and high‐grade tumors.^11^ Few researches have specifically focused on adults GGs.^3^, ^14^, ^21^, ^22^, ^23^ To our knowledge, this study, including 703 cases based on the SEER database, is the largest series of adult low‐grade GGs, and is the only study to compare several different treatment patterns (including surgery, and combined adjuvant RT and/or chemotherapy) simultaneously with a large number of adults. Additionally, our data identify that adjuvant treatment, especially the RT, may have a negative impact on adult patient's survival and prognosis.

### 4.1 Epidemiological and tumor characteristics

GGs generally has predilection in young adult population, especially in the second, third, or fourth decades of life.^4^ In our study, 64% of the patients were below the age of 40 years, and the mean and median ages at diagnosis were 36.0 and 32.0 years, respectively, which is consistent with the previously reported median age for adult low‐grade GGs of 27–40 years.^3^, ^14^, ^23^ Patients diagnosed with high‐grade GGs usually were older than those with low‐grade GGs.^22^, ^24^ Although the majority of studies showed a male predominance in either adult or pediatric population,^3^, ^15^, ^22^ the male‐to‐female ratio was close to 1:1 in our study, similar to some other studies.^13^, ^14^ Finally, the predominant tumor sites located in temporal and frontal lobes observed in the present study also concur with previously published series of low‐grade GGs or high‐grade GGs.^8^, ^11^, ^22^, ^24^ However, the proportion of temporal lobe location in pediatric patients could reach 47.4%, higher than adults.

In terms of treatment, GTR was performed in 54.7% of adult patients, which corresponds to the previous reports that the rate of GTR ranges from 47% to 72% in low‐grade GGs.^3^, ^8^, ^11^, ^14^, ^16^, ^21^, ^25^ For adjuvant therapies in our study, 6.0% and 2.1% of patients received RT and chemotherapy, respectively. The existing research data on RT and chemotherapy in adult patients with low‐grade GGs are limited. Yust‐Katz et al.^3^ reported that 29% of patients underwent RT and 8% of patients underwent chemotherapy in a total of 62 cases with low‐grade GGs, however, these two proportions in the other study including 181 cases were 3.9% and 1.7%, respectively.^14^

### 4.2 Factors associated with survival

Marital status and race were not crucial predictors of survival in both univariate and multivariate analysis. However, age at diagnosis, gender, tumor site, and treatment pattern were significant factors of survival in adult patients with low‐grade GGs. Although age was not identified as a prognostic factor in some published series, ^11^, ^17^, ^22^ our result showed that patients with age ≥40 years had a worse prognosis, similar to another study.^3^ There are few studies considering gender as a survival predictor, nevertheless, we found that a poorer prognosis in males, in consistent with the study by Rumana et al.^26^ Compared with other tumor sites, temporal lobe was not only the most common location of GGs, but also an important factor of survival in our study, associated with a significantly prolonged OS time, in agreement with the previous studies.^11^, ^22^ This may be due to the fact that the temporal lobe is relatively far away from important functional areas, and the tumor is easy to undergo GTR. In addition, patients with GTR had the highest 5‐ and 10‐year OS rates in this study, making it as a significantly prognostic indicator in predicting OS in adult low‐grade GGs, in line with other reported data, which might be due to the reduced risk of tumor recurrence and/or malignant transformation.^2^, ^3^, ^8^, ^11^, ^16^ Even with anaplastic GGs, GTR is also regarded as a predictor with improved outcomes.^24^, ^27^, ^28^, ^29^

### 4.3 Tumor management

Maximal safe tumor resection is the gold standard in the treatment of both low‐grade and high‐grade GGs. Our current results demonstrate that GTR is significantly better than STR in regard to OS. However, there were still 45.3% of patients who could not be completely resected, in which 11.3% of these patients underwent adjuvant treatment such as RT and/or chemotherapy again. The role of RT and chemotherapy in an adult population remains controversial up to now. Although a large retrospective meta‐analysis showed that postoperative RT could significantly improve local control in patients with GGs who underwent STR, the benefit on OS was not observed,^11^ and many other studies also reported that RT had little impact on progression‐free survival (PFS) or OS.^3^, ^8^, ^13^, ^14^, ^15^, ^16^ The important finding in our study shows that adjuvant RT may have a potentially adverse effect on survival in adult patients with low‐grade GGs. The same result is also found in patients underwent GTR. The concrete reason is unknown due to the limited clinical information included in the SEER database, however, according to some previously published data, it is most likely attribute to the side effects and increased risk of malignant progression after such adjuvant treatment.^3^, ^18^, ^26^

The study of chemotherapy as the adjuvant treatment in GGs is rare. Nevertheless, similar to RT, little influence was found on prognosis in these limited reports.^3^, ^14^, ^16^, ^22^ Our results demonstrate that adjuvant chemotherapy has no positive effect on OS in patients underwent whatever surgery. In a recent study, Lundar et al. suggested that repeat surgery should be considered before given the adjuvant therapy in patients with incomplete primary resection or recurrent GGs.^13^ Another study also advocated close observation without adjuvant treatment after surgery, even the tumor was located in brainstem.^30^ Therefore, given these significant findings and literatures analysis, we do not recommend to further take adjuvant RT and/or chemotherapy after surgery in adult patients with low‐grade GGs, unless the malignant transformation has been confirmed. Certainly, larger collaborative multi‐institutional prospective studies are still warranted to determine treatment consensus.

In addition, with the new era of advance targeted therapies and the frequent BRAF mutation in GGs, we also advocate such treatments options using BRAF inhibitors (e.g., vemurafenib or dabrafenib) and anticipate the promising results of the ongoing clinical trials in GGs.

### 4.4 Limitations

Although a large amount of invaluable demographic, diagnostic, and treatment data for rare brain tumors such as GGs can be found in the SEER database, there are still several limitations to the current study. First, there are no data available on tumor progression or recurrence rates, which are important to evaluate the patients PFS. Second, patients' quality of life and symptoms, such as neurologic sequelae and seizure control, which might relate to survival, are not recorded in the SEER database. Third, SEER does not provide details on the adjuvant treatments, such as dose and type of RT, chemotherapeutic agents and cycles, and the exact time of adjuvant treatments. Fourth, the number of patients receiving adjuvant treatments is small, which may have a certain impact on the result analysis. Fifth, it is unable to perform a pathological review of the tumors, which means that some tumors may have been misdiagnosed due to the low interobserver agreement in the diagnosis of different glioneuronal tumors.^31^, ^32^ In addition, there is a lack of molecular markers, such as BRAF V600E mutations, which do not allow us to investigate the relationship between markers and survival. Nevertheless, considering the prospective studies not available and not be expected in the near future, due to the rarity of GGs, a large retrospective study like this appears to be the best and most useful approach available to define the optimal treatment for these tumors. Therefore, despite several limitations, this is the largest published series and the best evidence available with respect to low‐grade GGs in adults up to date.

## 5 CONCLUSION

This is the largest retrospective study of adult low‐grade GGs, including 703 cases from the SEER database. Our data reaffirms three important prognostic factors such as low age, female gender, and a temporal lobe location. The extent of surgical resection is also still a positive predictor for OS, and patients should undergo GTR whenever safely possible. The use of adjuvant RT and/or chemotherapy may have a potentially negative impact on patient's survival and prognosis. Therefore, adjuvant RT and/or chemotherapy is not recommended to consider after whatever surgery in adult patients with low‐grade GGs, unless the malignant transformation has been confirmed.

## CONFLICT OF INTEREST DISCLOSURES

The authors have no conflicts of interest to declare.

## AUTHOR CONTRIBUTIONS

Xiaoning Lin: Study design, data collection, data analysis/interpretation, figure preparation, manuscript drafting, reviewing/editing manuscript. Rong Huang: Data collection, data analysis/interpretation, manuscript drafting, reviewing/editing manuscript. Pengfei Zhang: Data analysis/interpretation, manuscript drafting, reviewing/editing manuscript. Jin Sun: Data analysis/interpretation, reviewing/editing manuscript. Guijiang Dong: Data analysis/interpretation, reviewing/editing manuscript. Yanlin Huang: Study design, data collection, data analysis/interpretation, manuscript drafting, reviewing/editing manuscript. Xinhua Tian: Study design, data collection, data analysis/interpretation, manuscript drafting, reviewing/editing manuscript.

## Supporting information

Table S1

## Data Availability

The data in present study are available in the Surveillance, Epidemiology, and End Results (https://seer.cancer.gov).
