# Supplementary material for: Low‐grade gangliogliomas in adults: A population‐based study
Source: Cancer Med. 2020 Oct 27;10(1):416–23. doi: 10.1002/cam4.3577 (PMC7826489; doi:10.1002/cam4.3577)
Supplement: Supplementary file 1 — Table S1 [file CAM4-10-416-s001.docx]

**Table S1** The relationship between clinical characteristics and treatments

| **Characteristics** | **Surgery only** | **Surgery + AT** | ***P*-value** |
| --- | --- | --- | --- |
|  | **Number (%)** | **Number (%)** |  |
| Age (years) |  |  | < .001 |
| < 40 | 434 (96.4%) | 16 (3.6%) |  |
| ≥ 40 | 223 (88.1%) | 30 (11.9%) |  |
| Gender |  |  | 0.768 |
| Male | 328 (93.2%) | 24 (6.8%) |  |
| Female | 329 (93.7%) | 22 (6.3%) |  |
| Tumor site |  |  | 0.001 |
| Temporal lobe | 267 (97.8%) | 6 (2.2%) |  |
| Frontal lobe | 94 (91.3%) | 9 (8.7%) |  |
| Other sites | 296 (90.5%) | 31 (9.5%) |  |

**Abbreviations:** AT, adjuvant treatment.
